# Impact of a nurse-based intervention on medication outcomes in vulnerable older adults

**DOI:** 10.1186/s12877-018-0905-1

**Published:** 2018-09-06

**Authors:** Michael A. Steinman, Marcelo Low, Ran D. Balicer, Efrat Shadmi

**Affiliations:** 1 University of California, 3333 California St, San Francisco, CA 94118 USA; 2 San Francisco VA Health Care System, 4150 Clement St, Box 181G, San Francisco, CA 94121 USA; 3 Clalit Research Institute, Tel Aviv, Israel; 4 University of Haifa, Haifa, Israel

**Keywords:** Polypharmacy, Medication management, Primary care, Israel, Aged, Multimorbidity, Quality of care

## Abstract

**Background:**

Medication-related problems are common in older adults with multiple chronic conditions. We evaluated the impact of a nurse-based primary care intervention, based on the Guided Care model of care, on patient-centered aspects of medication use.

**Methods:**

Controlled clinical trial of the Comprehensive Care for Multimorbid Adults Project (CC-MAP), conducted among 1218 participants in 7 intervention clinics and 6 control (usual care) clinics. Inclusion criteria included age 45–94, presence of ≥3 chronic conditions, and Adjusted Clinical Groups (ACG) score > 0.19. The co-primary outcomes were number of changes to the medication regimen between baseline and 9 month followup, and number of changes to symptom-focused medications, markers of attentiveness to medication-related issues.

**Results:**

Mean age in the intervention group was 72 years, 59% were women, and participants used a mean of 6.6 medications at baseline. The control group was slightly older (73 years) and used more medications (mean 7.1). Between baseline and 9 months, intervention subjects had more changes to their medication regimen than control subjects (mean 4.04 vs. 3.62 medication changes; adjusted difference 0.55, *p* = 0.001). Similarly, intervention subjects had more changes to their symptomatic medications (mean 1.38 vs. 1.26 changes, adjusted difference 0.20, *p* = 0.003). The total number of medications in use remained stable between baseline and follow-up in both groups (*p* > 0.18).

**Conclusion:**

This nurse-based, primary care intervention resulted in substantially more changes to patients’ medication regimens than usual care, without increasing the total number of medications used. This enhanced rate of change likely reflects greater attentiveness to the medication-related needs of patients.

**Trial registration:**

This trial is registered at https://clinicaltrials.gov, trial number NCT01811173.

**Electronic supplementary material:**

The online version of this article (10.1186/s12877-018-0905-1) contains supplementary material, which is available to authorized users.

## Background

Prescribing for older adults with multiple chronic conditions often leads to medication regimens that are overly complicated, difficult to adhere to, and contain multiple drug-drug and drug-disease interactions [1, 2]. In addition, prescribing decisions often do not attend to patient preferences, abilities, and goals of care [3]. This leaves patients feeling sidelined and disengaged from their care, with medication regimens that are not tailored to their needs and preferences [4].

Addressing these patient-centered issues requires patient-centered approaches to improving medication use. Such strategies use an understanding of patients’ needs and abilities to inform treatment decisions [5]. In addition, these strategies can help patients adhere to and properly use medications to maximally benefit their health and achieve their goals [6]. Yet, this is easier said than done. Programs such as patient-centered medical homes are promising but have shown mixed results, and there are few models of care that consistently achieve these ends and are practical for widespread use [7].

In recent years, a promising new model called Guided Care has been proposed that is well suited to address these needs [8]. Guided Care is a system of comprehensive, interdisciplinary care that is tailored to the needs of older adults with multiple chronic conditions [9]. In this model, a registered nurse based in a primary care clinic works with a panel of vulnerable older patients. The nurse interfaces with these patients via home visits, over the telephone, and in the clinic. These interactions include elements of case management, support for patient self-management, and help with transitions of care. The nurse also works with patients’ clinicians to help coordinate care and bring patients’ needs, abilities, and preferences to clinical decision-making. Although it was not designed specifically to improve medication use, and does not incorporate any structured elements that focus explicitly on medications (such as medication review or reconciliation), its philosophy and processes are tightly aligned with the pharmaceutical care needs of older adults [10].

Previous studies have evaluated the impact of Guided Care on several outcomes such as mortality, mental and physical health, and caregiver burden [11–16]. However, little work has been done on how Guided Care impacts medication use and outcomes in vulnerable older adults. To fill this important evidence gap, we evaluated the effect of an intervention based on the Guided Care model on several aspects of medication prescribing for older adults in Israel. We hypothesized that older adults receiving this intervention would have improved markers of patient-centered prescribing – namely, more changes to their overall medication regimens, and more changes to their symptom-focused medications, representing greater attentiveness to adjusting medication regimens in response to patient symptoms, challenges with existing medications, and goals of care.

## Methods

### Intervention and study population

This study uses data from the Comprehensive Care for Multimorbid Adults Project (CC-MAP), a controlled clinical trial of a nurse-based intervention that was conducted within primary care clinics of Clalit Health Systems [17]. Clalit is Israel’s largest integrated health care provider and insurer, serving approximately half the country’s population.

In the CC-MAP study, representative (but not randomized) primary care clinics within 2 regions of Israel were selected as intervention and control sites. In the 7 intervention sites, nurses trained in the CC-MAP model were embedded in a clinic, which typically comprised 3 to 4 physicians plus support staff. CC-MAP nurses followed a panel of vulnerable adults who met the inclusion/exclusion criteria outlined below. Six control clinics were selected with similar demographic and health system characteristics as the intervention clinics. In the control clinics, patients received usual care.

Intervention patients met with the CC-MAP nurse monthly in person or by phone, and in person at least once per quarter, to review their care plan, make adjustments and receive counseling as needed, and follow up. When a patient was hospitalized, the CC-MAP nurse contacted the patient immediately after discharge to review changes in treatment recommendations, and as needed alert the primary care physician to implement treatment changes described in discharge recommendations. As this complex intervention was tailored to the needs and preferences of each participant, some subjects received more intensive services and contacts than others, although all intervention subjects enrolled had a minimum of 3 contacts (in person or by telephone) with CC-MAP nurses per year, with most having substantially more. There was no specific medication reconciliation or pharmacy component to the intervention, although since the nurse was part of a nurse-primary care physician team, part of her role was to periodically assess the patient’s status, including adherence to the care plan. Thus, when the nurse identified that the patient had side effects from medications, or that he/she was not adhering to the treatment, she notified the primary care physician. This provided an opportunity to be able to tailor care to patients’ evolving status and needs.

**Fig. 1:**
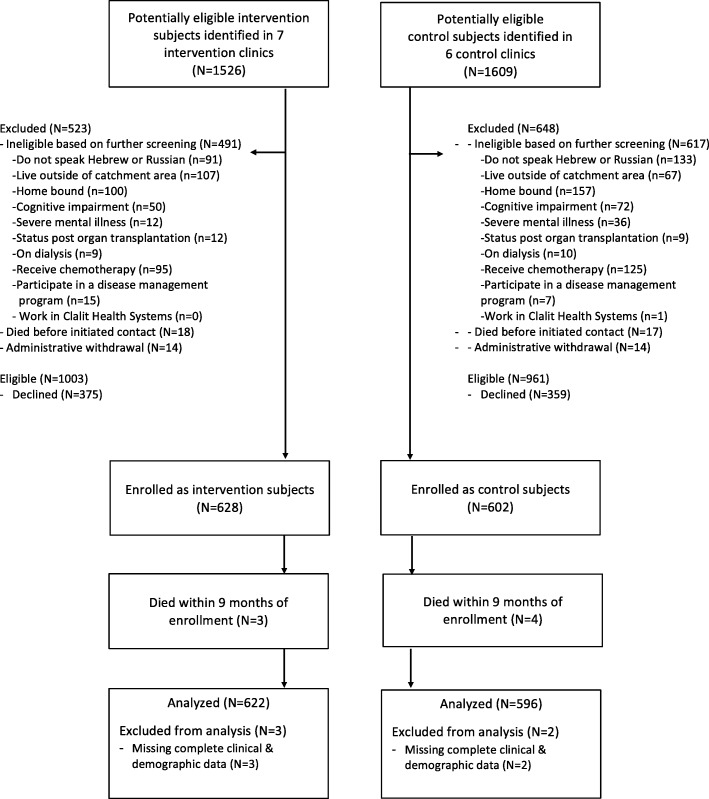
CONSORT diagram. People who died within the first 9 months after enrollment could not be analyzed since 9-month medication outcome data were not available for these subjects

Inclusion criteria included community-dwelling adults age 45–94 years and the presence of 3 or more chronic conditions. In addition, we employed a Johns Hopkins Adjusted Clinical Group® (ACG) system predictive modeling score using diagnosis and pharmacy data (the DxRxPM model), restricting enrollment to people with a score > 0.19, indicating high risk of poor clinical outcomes [18]. Exclusion criteria included inability to speak Hebrew or Russian, current participation in a disease management program, use of dialysis or chemotherapy, advanced dementia, or severe mental illness such as schizophrenia (see Fig. 1). Over 70% of the study population was age 65 and older. Intervention subjects were enrolled between April 2013 and June 2014, and control subjects between November 2013 and March 2015.

### Measures

The pre-specified primary outcome of the CC-MAP trial was hospital admissions for ambulatory-sensitive conditions, which the trial was powered to detect with an effect size of 0.3 and a type I error of 0.05. After the trial commenced but prior to reviewing outcomes data, we selected 2 new outcomes of interest related to medications, listed below. We chose these outcomes based on domains where we hypothesized the CC-MAP intervention could have particularly beneficial impacts on medication use. We considered these 2 outcomes co-primary for purposes of this study.

Medication use was assessed using pharmacy dispensing data from Clalit pharmacies and from non-Clalit pharmacies where Clalit insurance was billed. Together these account for the strong majority of prescription medications filled by Clalit members, who have powerful financial incentives to fill their medications using their Clalit benefits [19, 20]. We employed a variant of methods recommended by Lund et al. to identify prevalent medication use at time points of interest [21]. Because it can be difficult to ascertain dose and duration of medications that are delivered topically or locally, we restricted our analyses to medication classes that are delivered systemically and/or have systemic effects.

#### Outcome #1 - Number of changes in medication regimen

We hypothesized that the CC-MAP intervention would lead to more changes in medication regimens by improving communication about patient symptoms, goals, and problems with existing treatment regimens. Based on prior work, we defined medication changes as the sum of medication additions (medications present at 9 months that were not present at baseline), medication discontinuations (medications present at baseline that were no longer present at 9 months), therapeutic substitutions (a switch from one medication to another within the same drug class, defined at the 4th level of the Anatomic Therapeutic Classification [ATC] system), and dose changes (the same medication given at different daily doses at baseline vs. 9 months) [22].

#### Outcome #2 – Change in use of symptom-focused medications

We hypothesized that improved communication about patient symptoms would lead to more fine-tuning of symptom-focused medications. Our co-primary outcome was the number of changes in symptom-focused medications between baseline and 9 months. A secondary outcome was the total number of symptom-focused medications in use at 9 months. Methods for defining symptom-focused medications are described in Additional file 1: Appendix S1.

#### Other variables

Patient demographics were collected from Clalit data systems. Comorbid conditions were assessed using algorithms developed for Clalit clinical and claims data, for example using inpatient and outpatient diagnoses, pharmacy records, and laboratory test results. The unweighted sum of 27 chronic conditions defined a patient-level comorbidity score. ACG scores were calculated using clinical and claims data using standard methods employed by Clalit and validated for CC-MAP patient selection [23, 24]. Higher ACG scores predict a variety of poor clinical outcomes [24].

### Main analyses

Our analyses used an intention to treat framework. Overall, 45 intervention subjects and 1 control subject withdrew from participation in the intervention and/or followup assessments between baseline and 9 months, and 3 intervention subjects moved to a different clinic. (The differential drop-out rate is likely explained by burden and/or dislike of the intervention by intervention subjects, whereas control subjects had only usual care.) Although these subjects withdrew from participation or moved, we were able to assess their medication outcomes as medication use was measured using pharmacy claims data, which were generated irrespective of trial participation. Thus, these subjects were included in the final analysis according to their original treatment assignment.

All analyses employed mixed effects regression, using random effects to adjust for clustering of study subjects within physicians, and fixed effects to adjust for region. Clinic-level random effects were negligible after accounting for physician random effects. Outcomes analyses controlled for potential confounders including age, sex, comorbidity count, ACG DxRxPM score, number of medications present at baseline, and the number of medication changes that had occurred between 9 months before baseline and baseline (to account each subject’s pre-baseline rate of medication changes). We used interaction terms to evaluate potential effect modification among pre-specified subgroups defined by age, ACG predictive modeling score, and number of medications used at baseline.

The enrollment period for intervention subjects began and ended earlier than the enrollment period for control subjects, with only partial overlap. To evaluate if secular trends could have impacted our results, we assessed the impact of enrollment time on our medication outcomes in each treatment group. In these analyses, enrollment time was not associated with outcomes in either group (*p* > .15 for all).

### Exploratory analyses

We conducted an exploratory analysis to understand the relationship between our primary outcome measure (number of medication changes) and patient-centeredness. We hypothesized that a greater number of medication changes over time would be associated with greater patient-centeredness, reflecting increased attention to patients’ medication-related needs and challenges. This analysis used data from the Patient Assessment of Chronic Illness Care (PACIC) questionnaire, which was completed by control subjects at baseline. (Accurate baseline data were not available for intervention subjects). Our predictor variable was the number of medication changes in the 9 months prior to the time the PACIC was administered. Our outcome variable was the sum of scores for the first 3 items on the PACIC questionnaire, which address the patient-centeredness of treatment planning, and which have been validated to have strong psychometric properties (Cronbach’s alpha 0.82, standardized factor loadings > 0.75) [25]. Answers to these items were summed into a score ranging from 3 (never involved in treatment planning) to 15 (always involved in treatment planning). We used Poisson regression to evaluate the predictor-outcome relationship, while controlling for potential confounders including number of medications in the pre-baseline period, number of chronic conditions, ACG predictive modeling score, age, race, and sex, and adjusting for clinician as a random effect (see Additional file 1: Appendix S5).

This study adheres to CONSORT guidelines and was approved by the institutional review boards of Clalit Health Services and the University of California, San Francisco.

## Results

Subject enrollment and follow-up data are shown in Fig. 1. Of 1230 subjects enrolled, 1218 had full data available for analysis at 9 month follow-up. The 622 intervention patients in the final analytic sample received care in 7 intervention clinics by 21 primary care physicians. The 598 control patients received care in 6 control clinics predominantly by 17 primary care physicians.

Baseline characteristics of intervention control patients are shown in Table 1. Compared with control patients, intervention patients were slightly younger, had fewer chronic conditions, and received fewer medications.

**Table 1:** Baseline characteristics of intervention and control subjects

	Intervention *N* = 622	Control *N* = 596
Age (mean, SD)	71.6 (10.2)	73.4 (9.9)
Female sex	365 (59)	329 (55)
No. of chronic conditions (mean, SD)^a^	4.1 (2.1)	4.8 (2.3)
ACG score (median, IQR)^b^	0.29 (0.22–0.42)	0.25 (0.18–0.39)
Number of medications at baseline (mean, SD)	6.6 (3.3)	7.1 (3.2)
Number of changes in medications in the 9 months prior to baseline	3.9 (2.7)	3.9 (2.8)
Number of symptom-focused medications at baseline		
0	203 (33)	150 (25)
1	161 (26)	165 (28)
2 or more	258 (41)	281 (47)

*p* value < 0.05 for difference between groups for age, number of chronic conditions, ACG score, and number of medications (overall and symptom-focused) at baseline

^a^ From a list of 27 chronic conditions

^b^ ACG scores for some subjects are below the enrollment threshold because these scores shifted between the time these subjects were identified as eligible to participate to the time they were enrolled

### Effect of CC-MAP intervention on changes in medication use

Between baseline and 9 months, intervention patients had a mean of 4.04 changes to their medication regimens (Table 2). Control patients had a mean of 3.62 changes. After adjusting for baseline characteristics, intervention patients had a mean of 0.55 more medication changes than control subjects (*p* = .001; see Additional file 1: Appendix S3 for additional information).

**Table 2:** Number of medication changes between baseline and 9 months, intervention vs. control group

	Intervention *N* = 622	Control *N* = 596	Adjusted difference in number of medication changes^a^	*p* value
Total number of changes to medication regimen (mean, SD)	4.04 (2.8)	3.62 (2.7)	0.55	.001
Total number of changes of symptom-focused medications (mean, SD)	1.38 (1.5)	1.27 (1.5)	0.20	.003

^a^ Adjusted for baseline subject characteristics and clustering

A similar pattern was observed for changes in the use of symptom-focused medications. Between baseline and 9 month followup, intervention patients had more changes to their regimen of symptom-focused medications than control patients (mean 1.38 vs. 1.27 changes, adjusted mean difference 0.20 changes, *p* = .003).

As a sensitivity analysis, we expanded our definition of symptom-focused medications to include drugs used to treat intermittent but highly symptomatic conditions, and medications with mixed symptom-focused and non-symptom-focused uses. Results were similar using this expanded definition: intervention patients had more changes to the regimen of symptom-focused medications than control patients (mean 1.89 vs 1.81 changes, adjusted mean difference 0.20 changes, *p* = .01).

### Characteristics of medication changes

In both the intervention and control groups, there was little difference in the total number of medications subjects used at baseline and at 9 months. The intervention group used a mean of 0.09 more medications at 9 months compared with baseline (*p* = 0.32 for change). The control group used a mean of 0.12 fewer medications at 9 months compared with baseline (*p* = 0.18 for change; *p* = .12 for difference in change between intervention and control groups).

The total number of symptom-focused medications also remained generally stable from baseline to 9 months, although there was a small, statistically significant increase in the intervention group. The intervention group used a mean of 0.13 more symptom-focused medications at 9 months compared with baseline (*p* = 0.02 for change). The control group used 0.03 fewer symptom-focused medications at 9 months (*p* = 0.59 for change; *p* = 0.06 for difference in number of changes between groups).

The distribution of types of medication changes (e.g. additions, discontinuations, therapeutic substitutions, and dose changes) were generally similar across groups (Table 3). The distribution of which classes of drugs were changed was also similar in the two groups. The most commonly changed medication class was cardiovascular medications, which accounted for 30% of changes in the intervention group and 29% in the control group (*p* = 0.31 for difference; see Additional file 1: Appendix S4 for additional information).

**Table 3:** Types of medication changes between baseline and 9 months, intervention vs. control group

Types of medication change	Intervention *N* = 622 Mean # of changes (% of total)^a^	Control *N* = 596 Mean # of changes (% of total)^a^	Adjusted difference^b^	*p* value
Medication additions	1.79 (44%)	1.51 (42%)	2%	0.37
Medication discontinuations	1.70 (42%)	1.63 (45%)	3%	0.09
Therapeutic substitutions	0.15 (4%)	0.15 (4%)	0%	0.91
Dose increases	0.18 (5%)	0.19 (6%)	1%	0.39
Dose reductions	0.21 (6%)	0.15 (4%)	2%	0.007
TOTAL NUMBER OF CHANGES	4.04 (100%)	3.62 (100%)	–	–

^a^ Results show the mean number of each type of medication change within each group, and the percent of all changes attributable to each type of change

^b^ Difference between intervention and control group in the percent of changes attributable to each type of change. Adjusted for baseline subject characteristics and clustering

### Subgroup effects

The impact of the intervention on both medication change outcomes did not vary between patients of different age (under 75 vs. 75 or more years), ACG score (under 0.27 vs. 0.27 or greater), number of medications at baseline (under 7 vs 7 or more), and number of symptom-focused medications at baseline (0 to 1 vs. 2 or more), with *p* values for interaction > 0.37 in each comparison.

### Exploratory analyses

In an exploratory analysis, we used data from the control group to evaluate the relationship between number of medication changes and the patient-centeredness of treatment planning. On a scale of 3–15 (with 15 being best), the median 3-item PACIC score was 6 (interquartile range, 3–9). There was a non-linear relationship between number of medication changes and 3-item PACIC scores. Compared to subjects with 0–2 medication changes, 3-item PACIC scores were a mean of 0.30 points higher in subjects with 3–5 medication changes, but 0.44 points lower in subjects with 6 or more changes (*p* = 0.003 for difference in scores between categories). After controlling for several potential confounders, the differences remained (*p* = .03; see Additional file 1: Appendix S5 for details).

## Discussion

In this controlled clinical trial, the CC-MAP intervention – a nurse based, primary care intervention based on the Guided Care model of care – improved several aspects of prescribing in vulnerable Israeli adults. Compared with usual care, patients receiving the CC-MAP intervention had more changes to their medication regimens in general, and more changes in their symptom-focused medications. This increased rate of changes may reflect more attentive management to patients’ medication-related needs.

Our findings build on prior research on the Guided Care model of care. Past studies have shown that Guided Care improves patient-centered processes of care, including goal-setting, coordination of care, decision support, and patient activation [11–14]. This study adds a new dimension to these findings by improving markers of patient-centered medication management. However, despite benefits in processes of care, a large trial of Guided Care did not demonstrate significantly beneficial effects on health outcomes including self-reported measures of physical and mental health, mortality, and several forms of health services utilization [15, 16]. Although these findings are disappointing, they are not the last word. The trial was underpowered to detect small but clinically meaningful differences in these outcomes, and provided lessons about potential future improvements in this care model [15, 16]. Subsequent work testing refined models of Guided Care are underway, including the CC-MAP trial, which is showing promise for improving these hard outcomes.

One noteworthy aspect of our findings is that improving medication use was not a primary goal of the intervention, and in fact the CC-MAP program had no structured elements that focused explicitly on medications. Rather, the improved markers of medication use that we observed appear to be a beneficial “side effect” of the more general goals of the CC-MAP program such as improving communication and care coordination. Vulnerable older adults require support in many areas, for example improving pharmacotherapy, assessing and maintaining functional and cognitive abilities, and much more [26]. Yet, it is impractical to offer multiple discrete interventions, each targeted to only one area [27]. In being able to improve a domain of care that was not an explicit focus of the intervention, CC-MAP and Guided Care show promise as a single intervention which that can favorably affect multiple domains.

The first two measures of prescribing that we evaluated – the number of changes in medications overall, and the number of changes in symptom-focused medications – are not standard measures of prescribing quality, and have not been validated as markers of this construct. Thus, we cannot be sure that the higher number of changes observed in the intervention group represents better care. Our analyses between number of medication changes and treatment-planning components of the PACIC score suggest there may be a threshold effect: a moderate number of changes are associated with more patient-centered care, but a large volume of changes has the opposite effect. However, this finding should be considered preliminary, since it used only a subset of the PACIC score and was unable to control for potentially important confounders.

Although these caveats are important, these markers nonetheless have potential to be valuable [22]. Many commonly used markers of prescribing quality, such as prescribing of drugs to avoid in older adults (e.g. the Beers and STOPP criteria) are not particularly patient-centered [28, 29]. What often matters most for patients is individualizing their medication regimen to suit their particular circumstance, yet studies have shown that clinical inertia and competing demands often prevent appropriate modification of regimens to meet changing patient needs [30–32]. Although more changes are not always better, in this context they may reflect more attention to individual patient circumstances [33].

Additional findings also shed light on the meaning of the increased rate of medication changes observed in the intervention group. The total number of medications used by intervention subjects remained stable despite the higher number of medication changes. The intervention thus appears to have enhanced “fine-tuning” of medications rather than adding to the already-large numbers of medications used by study subjects. It is also noteworthy that the intervention did not preferentially affect one type of medication change or one class of medications. This appears to reflect a generalized effect of the intervention rather than focused changes in one specific area.

Our study has several limitations. Due to practical considerations the selection of intervention and control clinics was not random. However, we were able to control for multiple baseline characteristics of subjects, including their pre-baseline rate of medication changes, which substantially reduces potential for bias in our results. Medication use was ascertained using pharmacy dispensing records. As a result, our measures of medication use are affected both by what medications the physician(s) ordered and patients’ adherence to obtaining those medications; we are unable to distinguish the relative contribution of each. Nonetheless, this does not affect our conclusions since both physician prescribing and patient adherence are important facets of medication use that may be improved by the intervention. In addition, we were unable to account for medications not captured in Clalit databases. However, this likely accounts for only a small fraction of medications filled, given strong incentives to fill medications using Clalit insurance benefits, and is unlikely to differ between intervention and control subjects. Finally, we did not evaluate the impact of the intervention on clinical outcomes such as medication errors, hospitalizations, or symptom control; these will be important avenues for future research.

## Conclusions

The CC-MAP intervention, a nurse-based primary care program based on the Guided Care model of care, improved markers of patient-centered prescribing in vulnerable adults. With further attention to medication-related issues, this program – which had little explicit focus on medication use – might further improve prescribing and improve medication-related outcomes in vulnerable older adults.

## Additional file

Additional file 1: **Appendix S1.** Defining symptom-focused medications – introduction. **Appendix S2.** Defining symptom-focused medications – drug class coding. **Appendix S3.** Full multivariable model comparing medication change outcomes in intervention and control subjects. **Appendix S4.** Distribution of medication classes used at baseline, and of medication classes that were changed between baseline and 9 months. **Appendix S5.** Relationship between number of medication changes and patient-centeredness of treatment planning (3-item PACIC score), in control group. (DOCX 53 kb)

## Abbreviations

ACG: Adjusted Clinical Group
CC-MAP: Comprehensive Care for Multimorbid Adults Project
PACIC: Patient Assessment of Chronic Illness Care
