# Supplementary material for: Impact of a nurse-based intervention on medication outcomes in vulnerable older adults
Source: BMC Geriatr. 2018 Sep 6;18:207. doi: 10.1186/s12877-018-0905-1 (PMC6127952; doi:10.1186/s12877-018-0905-1)
Supplement: Supplementary file 1 — Appendix S1. Defining symptom-focused medications – introduction. Appendix S2. Defining symptom-focused medications – drug class coding. Appendix S3. Full multivariable model comparing medication change outcomes in intervention and control subjects. Appendix S4. Distribution of medication classes used at baseline, and of medication classes that were changed between baseline and 9 months. Appendix S5. Relationship between number of medication changes and patient-centeredness of treatment planning (3-item PACIC score), in control group. (DOCX 53 kb) [file 12877_2018_905_MOESM1_ESM.docx]

**Additional file 1**

Appendix S1: Defining symptom-focused medications - introduction

Appendix S2: Defining symptom-focused medications – drug class coding

Appendix S3: Full multivariable model comparing medication change outcomes in intervention and control subjects

Appendix S4: Distribution of medication classes used at baseline, and of medication classes that were

changed between baseline and 9 months

Appendix S5: Relationship between number of medication changes and patient-centeredness of treatment planning (3-item PACIC score), in control group.

**Appendix S1: Defining Symptom-Focused Medications - Introduction**

There is no universally-accepted method for identifying which medications are primarily oriented toward controlling symptoms (e.g. symptom control medications). For this project, we reviewed existing definitions and coding schemas. Finding none of these suitable for our needs, we created a new coding schema that borrowed from this prior work.

First, we based our definition of symptom-control medications on the definition of symptomatic treatment listed in Stedman’s medical dictionary: “Therapy aimed at relieving symptoms without necessarily affecting the basic underlying cause(s) of the symptoms.” To establish a more concrete definition, we defined a medication as being a symptom-control medication if, in most uses, it is likely to improve symptoms within 30 days and is typically prescribed mainly for that purpose. This includes medications that do not affect the underlying physiology or prognosis of a disease, and those that do while also providing meaningful symptomatic relief in the short term. We set the 30 day criterion because it (1) is a reasonably near-term goal, while allowing for inclusion of drugs intended to treat symptoms that have a delayed onset of action, (2) comports with clinical judgment around medications such as antidepressants and antiepileptic drugs used for neuropathic pain, which treat the symptoms for conditions such as pain and depression that we wish to include; (3) is short enough that it does not have extensive overlap with medications used for long-term disease modification or prevention.

We did not include medications administered topically or given locally for ophthalmologic, otic, oral or naval cavity, or peri-anal conditions. This is because these medications can often be difficult to divide into symptom-focused vs other purposes, and because they are often prescribed by specialists, who were not the primary focus of our research. For similar reasons, we did not consider chemotherapeutic agents. We also excluded antiinfective agents because it is difficult to disentangle their symptom-focused from disease-control benefits, and because they are not used for chronic conditions that are the focus of our research. We did include medications administered via gastrointestinal, transdermal, and pulmonary routes (e.g. suppositories, systemic drugs given via transdermal systems such as rivastigmine and fentanyl, and inhalers for lung disease).

Using these definitions, we classified all medications into one of four categories:

1. Symptom-focused medications
   - For example, medications use to treat pain or constipation
2. Medications used to prevent future flare-ups of episodic conditions that are highly symptomatic but otherwise do not affect longevity or need for hospitalization
   - For example, medications used to prevent flare-ups of gout and biliary colic.
3. Medications that are on the borderline of being considered symptom-focused, such as medications often used long-term to affect the underlying physiology of a disease but which may also have some short-term symptomatic benefits
   - For example, oral corticosteroids
4. Not a symptom-focused medication
   - For example, most antihypertensive medications

Our primary analysis used the narrow definition of symptom-focused medications, i.e. “A” above. In sensitivity analyses, we expanded the definition to also include medications in categories “B” and “C.”

Some medications can be used for more than one purpose. In these cases, we classified the medication based on clinical judgment about what are likely to be its most common uses.

To ease the burden of coding, we classified medications at the 3^rd^ level of Anatomic Therapeutic Classification (ATC) coding system, which is a moderately specific level of classification (e.g. ATC code C08C, “Selective calcium channel blockers with direct cardiac effects”, or N04B, ,”Anti-Parkinson drugs – dopaminergic agents”). Where there was a mix of symptom-focused and non-symptom-focused medications within a given 3^rd^ level classification, we coded it based on the status of drugs in that class that according to the opinion of the coders were most commonly used.

Two reviewers (Drs. Steinman and Roitman) independently classified all medications. Where their initial ratings disagreed, they discussed the differences to reach consensus. Where consensus could not be reached, we consulted a 3^rd^ physician (Alexander Smith, MD, MPH) to adjudicate the final decision.

By means of comparison, previous work in this area by Currow et al, Sera et al, and the International Association for Hospice and Palliative Care (IAHPC) has classified medications as symptom-focused or non-symptom focused (“chronic”). Medications that may be controversial or which received different status in different lists are listed below; this table does not include medications on which all 3 existing lists agreed. In this table, “YES” indicates that the drug is considered a symptom-focused medication by the list; “NO” indicates it is NOT considered a symptom-focused medication, and “—“ indicates that the drug was not mentioned in the list either as a symptom-focused or non-symptom-focused medication.

| Medication | List | | |
| --- | --- | --- | --- |
|  | Currow | Sera | IAHPC |
| Corticosteroids  Corticosteroids in general  Dexamethasone  Prednisolone | YES  YES  NO | YES  --  - | --  YES  YES |
| Antiepileptics  Antiepileptics in general  Gabapentin  Carbamazepine | --  --  -- | NO  --  -- | --  YES  YES |
| Acid-reducers  Acid-reducers in general  Proton-pump inhibitors  H2 blockers | --  NO NO | NO  --  -- | --  --  -- |
| Diuretics | -- | NO | -- |

Currow DC, Stevenson JP, Abernethy AP, Plummer J, Shelby-James TM. Prescribing in palliative care as death approaches. J Am Geriatr Soc. 2007 Apr;55(4):590-5. PubMed PMID: 17397439.

Sera L, McPherson ML, Holmes HM. Commonly prescribed medications in a population of hospice patients. Am J Hosp Palliat Care. 2014 Mar;31(2):126-31.

International Association for Hospice and Palliative Care (IAHPC) Essential Medications List (via http://hospicecare.com/uploads/2011/8/iahpc-essential-meds-en.pdf)

**Appendix S2: Defining Symptom-focused Medications – Drug Class Coding**

Medications were coded at the 3^rd^ level of the Anatomic Therapeutic Classification (ATC) system.

**Table A2-1: Symptom-focused Medications**

| **ATC Code** | **Description** |
| --- | --- |
| A02A | ANTACIDS |
| A02X | OTHER DRUGS FOR ACID RELATED DISORDERS |
| A03A | DRUGS FOR FUNCTIONAL GASTROINTESTINAL DISORDERS |
| A03B | BELLADONNA AND DERIVATIVES, PLAIN |
| A03C | ANTISPASMODICS IN COMBINATION WITH PSYCHOLEPTICS |
| A03D | ANTISPASMODICS IN COMBINATION WITH ANALGESICS |
| A03E | ANTISPASMODICS AND ANTICHOLINERGICS IN COMBINATION WITH OTHER DRUGS |
| A03F | PROPULSIVES |
| A04A | ANTIEMETICS AND ANTINAUSEANTS |
| A06A | DRUGS FOR CONSTIPATION |
| A07B | INTESTINAL ADSORBENTS |
| A07D | ANTIPROPULSIVES |
| A07F | ANTIDIARRHEAL MICROORGANISMS |
| A07X | OTHER ANTIDIARRHEALS |
| G04B | UROLOGICALS |
| G04C | DRUGS USED IN BENIGN PROSTATIC HYPERTROPHY |
| M01A | ANTIINFLAMMATORY AND ANTIRHEUMATIC PRODUCTS, NON-STEROIDS |
| M03A | MUSCLE RELAXANTS, PERIPHERALLY ACTING AGENTS |
| M03B | MUSCLE RELAXANTS, CENTRALLY ACTING AGENTS |
| M03C | MUSCLE RELAXANTS, DIRECTLY ACTING AGENTS |
| N02A | OPIOIDS |
| N02B | OTHER ANALGESICS AND ANTIPYRETICS |
| N02C | ANTIMIGRAINE PREPARATIONS |
| N03A | ANTIEPILEPTICS |
| N04A | ANTICHOLINERGIC AGENTS |
| N04B | DOPAMINERGIC AGENTS |
| N05A | ANTIPSYCHOTICS |
| N05B | ANXIOLYTICS |
| N05C | HYPNOTICS AND SEDATIVES |
| N06A | ANTIDEPRESSANTS |
| N06B | PSYCHOSTIMULANTS, AGENTS USED FOR ADHD AND NOOTROPICS |
| N06C | PSYCHOLEPTICS AND PSYCHOANALEPTICS IN COMBINATION |
| N07C | ANTIVERTIGO PREPARATIONS |
| R01B | NASAL DECONGESTANTS FOR SYSTEMIC USE |
| R03A | ADRENERGICS, INHALANTS |
| R03B | OTHER DRUGS FOR OBSTRUCTIVE AIRWAY DISEASES, INHALANTS |
| R03C | ADRENERGICS FOR SYSTEMIC USE |
| R03D | OTHER SYSTEMIC DRUGS FOR OBSTRUCTIVE AIRWAY DISEASES |
| R05C | EXPECTORANTS, EXCL. COMBINATIONS WITH COUGH SUPPRESSANTS |
| R05D | COUGH SUPPRESSANTS, EXCL. COMBINATIONS WITH EXPECTORANTS |
| R05F | COUGH SUPPRESSANTS AND EXPECTORANTS, COMBINATIONS |
| R05X | OTHER COLD PREPARATIONS |
| R06A | ANTIHISTAMINES FOR SYSTEMIC USE |
| R07A | OTHER RESPIRATORY SYSTEM PRODUCTS |

**Table A2-2: Medications used to prevent episodic symptomatic conditions**

| **ATC Code** | **Description** |
| --- | --- |
| A05A | BILE THERAPY |
| A05C | DRUGS FOR BILE THERAPY AND LIPOTROPICS IN COMBINATION |
| M04A | ANTIGOUT PREPARATIONS |

**Table A2-3: “Borderline” medications - medications often used long-term to affect the underlying physiology of a disease but which may also be used to provide near-term symptomatic benefits**

| **ATC Code** | **Description** |
| --- | --- |
| A02B | DRUGS FOR PEPTIC ULCER AND GASTRO-OESOPHAGEAL REFLUX DISEASE |
| A07C | ELECTROLYTES WITH CARBOHYDRATES |
| A07E | INTESTINAL ANTIINFLAMMATORY AGENTS |
| A09A | DIGESTIVES, INCL. ENZYMES |
| A14A | ANABOLIC STEROIDS |
| A14B | OTHER ANABOLIC AGENTS |
| C03C | HIGH-CEILING DIURETICS |
| C03X | OTHER DIURETICS |
| C04A | PERIPHERAL VASODILATORS |
| G03B | ANDROGENS |
| G03C | ESTROGENS |
| H02A | CORTICOSTEROIDS FOR SYSTEMIC USE, PLAIN |
| H02B | CORTICOSTEROIDS FOR SYSTEMIC USE, COMBINATIONS |
| H03A | THYROID PREPARATIONS |
| L04A | IMMUNOSUPPRESSANTS |
| M01B | ANTIINFLAMMATORY/ANTIRHEUMATIC AGENTS IN COMBINATION |
| M09A | OTHER DRUGS FOR DISORDERS OF THE MUSCULO-SKELETAL SYSTEM |
| N06D | ANTI-DEMENTIA DRUGS |
| N07B | DRUGS USED IN ADDICTIVE DISORDERS |
| L03 | IMMUNOSTIMULANTS |
| L04 | IMMUNOSUPPRESSANTS |

**Appendix S3: Full multivariable models comparing medication change outcomes in intervention and control subjects**

**Table A3-1: Impact of CC-MAP intervention on number of changes to the medication regimen between baseline and 9 months follow-up.**

| **Subject characteristics** | **Adjusted difference between groups**  **(Beta coefficient with 95% CI)** | **P value** |
| --- | --- | --- |
| **Primary predictor:** |  |  |
| Study group  Control  Intervention | --  0.55 (0.24 to 0.86) | <0.001 |
| **Control variables:** |  |  |
| Age | 0.00 (-0.01 to 0.01) | 0.96 |
| Female sex | 0.26 (0.00 to 0.53) | 0.05 |
| No. of chronic conditions | 0.08 (0.01 to 0.15) | 0.02 |
| ACG score | 0.31 (-0.45 to 1.08) | 0.42 |
| Number of medications at baseline | 0.20 (0.15 to 0.25) | <0.001 |
| Number of medication changes between 9 months *before* baseline to baseline | 0.36 (0.31 to 0.42) | <0.001 |

Model also adjusts for clustering structure and region

** Continuous predictors (age, number of chronic conditions, ACG score, number of medications at baseline, and number of medication changes between baseline and 9 months prior) measured by 1-unit increments

**Table A3-2: Impact of CC-MAP intervention on number of changes in *symptom-focused* medications between baseline and 9 months follow-up.**

| **Subject characteristics** | **Adjusted difference between groups**  **(Beta coefficient with 95% CI)** | **P value** |
| --- | --- | --- |
| **Primary predictor:** |  | **P** |
| Study group  Control  Intervention | --  0.20 (0.07 – 0.33) | 0.003 |
| **Control variables:** |  |  |
| Age | 0.00 (-0.01 to 0.01) | 0.82 |
| Female sex | 0.03 (-0.10 to 0.17) | 0.61 |
| No. of chronic conditions | 0.01 (-0.02 to 0.04) | 0.59 |
| ACG score | -0.04 (-0.42 to 0.34) | 0.82 |
| Number of symptom-focused medications at baseline | 0.33 ( 0.28 to 0.37) | <0.001 |
| Number of changes in symptom-focused medications between 9 months *before* baseline to baseline | 0.38 (0.33 to 0.44) | <0.001 |

Model also adjusts for clustering structure and region

** Continuous predictors (age, number of chronic conditions, ACG score, month of enrollment, number of symptom-focused medications at baseline, and number of symptom-focused medication changes between baseline and 9 months prior) measured by 1-unit increments

**Appendix S4: Distribution of medication classes used at baseline, and of medication classes that were changed between baseline and 9 months**

| ATC drug class code | Drug class description | Percent of total medications used at baseline | | |  | Percent of medication changes between baseline and 9 months | | |
| --- | --- | --- | --- | --- | --- | --- | --- | --- |
|  |  | **Intervention** | **Control** | **P value*** |  | **Intervention** | **Control** | **P value *** |
| A | Gastrointestinal & metabolic | 22% | 21% | 0.11 |  | 21% | 23% | 0.13 |
| B | Blood / hematologic | 5% | 5% | 0.33 |  | 5% | 5% | 0.29 |
| C | Cardiovascular | 42% | 41% | 0.34 |  | 30% | 29% | 0.31 |
| G | Genitourinary & reproductive | 4% | 4% | 0.69 |  | 5% | 4% | 0.96 |
| H | Endocrinologic & hormonal (ex. sex hormones & insulins) | 3% | 3% | 0.23 |  | 3% | 4% | 0.01 |
| J | Antiinfectives (systemic) | 2% | 1% | 0.001 |  | 6% | 3% | <0.001 |
| L | Antineoplastic & immunomodulating | 1% | 1% | 0.005 |  | 1% | 1% | 0.06 |
| M | Musculoskeletal | 4% | 4% | 0.67 |  | 5% | 5% | 0.78 |
| N | Nervous system | 13% | 15% | 0.06 |  | 17% | 18% | 0.91 |
| P | Anti-parasitic | 0.2% | 0.3% | 0.28 |  | 0.4% | 0.5% | 0.53 |
| R | Respiratory | 4% | 4% | 0.71 |  | 7% | 6% | 0.72 |
| V | Miscellaneous other | 0.0% | 0.1% | 0.14 |  | 0.1% | 0.2% | 0.44 |
| TOTAL |  | 100% | 100% |  |  | 100% | 100% |  |

* Adjusted for baseline subject characteristics and clustering

Drug classes primarily related to topical and locally-active medications (e.g. dermatological medications, opthalmological medications, etc) are not listed because analyses were restricted to medication classes that predominantly have systemic effects.

**Appendix S5: Relationship between number of medication changes and patient-centeredness of treatment planning (3-item PACIC score), in control group.**

The PACIC questionnaire contains 20 items that cover a wide range of topics. To focus on the patient-centeredness of treatment (e.g. medication) planning, we evaluated only the first 3 items, which inquire about whether the patient was asked to talk about problems with their medications, was asked for ideas during treatment planning, and was given choices about treatments to consider. In validation work, factor analyses demonstrated that the items each address the concept of patient engagement (Taggart J et al, *J Eval Clin Pract.* 2011;17(2):215-221). Following the PACIC scoring system, we summed response to these items to generate a score ranging from 3 (never involved in treatment planning) to 15 (always involved in treatment planning).

Median value of the 3-item PACIC in study population was 6, interquartile range 3-9.

|  | **Bivariate analysis** | | **Multivariable analysis** | |
| --- | --- | --- | --- | --- |
|  | IRR  (95% CI) | P value for within-group difference | IRR  (95% CI) | P value for within-group difference |
| Number of medication changes  0 – 2  3 – 5  6 or more | ref  1.08 (0.98 to 1.20)  0.88 (0.78 to 1.00) | 0.003 | ref  1.08 (0.97 to 1.20)  0.92 (0.80 to 1.05) | 0.03 |
| Number of medications at pre-baseline  0 – 4  5 – 8  9 or more |  |  | ref  1.09 (0.97 to 1.23)  0.92 (0.80 to 1.07) | 0.01 |
| Age (per 10 years) |  |  | 0.95 (0.90 to 0.99) | 0.02 |
| Female sex |  | 1 | 1.03 (093 to 1.13) | 0.59 |
| No. of chronic conditions |  |  | 0.98 (0.96 to 1.01) | 0.20 |
| ACG score †  0.00 – 0.19  0.20 – 0.29  0.30 or more |  |  | ref  1.00 (0.89 to 1.13)  1.08 (0.96 to 1.21) | 0.31 |

* IRR = incident rate ratio, the measure of association expressed in Poisson regression. Variables assessed at study baseline, except number of medications at pre-baseline (9 months before baseline) and number of medication changes between pre-baseline period and baseline.

† ACG scores vary over time since they are calculated using data from a defined period prior to the date of assessment. Although only subjects with ACG score >0.19 at the time of screening were included in the study, some of these subjects’ ACG scores were lower on the date of enrollment (study baseline).

Taggart J, Chan B, Jayasinghe UW, et al. Patients Assessment of Chronic Illness Care (PACIC) in two Australian studies: structure and utility. *J Eval Clin Pract.* 2011;17(2):215-221.
